# Upgrading from a Dual-chamber Pacemaker to a Cardiac Resynchronization Therapy Defibrillator in Situs Inversus Totalis with Dextrocardia Following Mitral Valve Replacement and Tricuspid Annuloplasty Using a Left-sided Approach

**DOI:** 10.19102/icrm.2021.120101

**Published:** 2021-01-15

**Authors:** Amato Santoro, Elodi Bacci, Claudia Baiocchi

**Affiliations:** ^1^Division of Cardiology, Azienda Ospedaliera Universitaria Senese, Siena, Italy

**Keywords:** Cardiac resynchronization therapy defibrillator, dextrocardia, situs inversus totalis

## Abstract

A 71-year-old female patient was referred to our center to upgrade a dual-chamber pacemaker to a cardiac resynchronization therapy defibrillator (CRT-D) following the detection of worsened systolic function (ejection fraction:25%–30%) via transthoracic echocardiography. The patient had situs inversus totalis with dextrocardia. She had undergone mitral valve replacement and tricuspid annuloplasty in July 2019, with a concomitant left upper pulmonary lobectomy for neoplasm, detected at cardiac tomography incidentally. In January 2020, we performed an upgrade of the preexisting device to a CRT-D system because the patient developed heart failure, reduction in systolic function, and numerous nonsustained ventricular tachycardias. The right ventricular lead that had been previously implanted was extracted. To facilitate the intervention, we decided to flip the fluoroscopic image, obtained with a right-anterior oblique view, by 180° (right–left), creating the optical impression of a levocardial position.

## Introduction

Situs inversus totalis (SIT) is a congenital condition in which the major visceral organs are reversed or mirrored from their normal positions. SIT associated with dextrocardia (DXC) is a rare congenital disease found in approximately one in 12,000 live births.^1^ DXC refers to a condition in which the heart is located on the right side of the chest. The most common and familiar form of DXC is classic mirror-image DXC, in which the anterior–posterior relationships of the various parts of the heart are normal but their right-to-left orientation is reversed. This condition is commonly associated with the liver shadow on the left and the stomach bubble on the right on X-ray films constituting the box of SIT.^2^ This report describes the procedure of upgrading from a dual-chamber pacemaker to a cardiac resynchronization therapy defibrillator (CRT-D) system in a patient with SIT and DXC suffering from heart failure.

## Case presentation

A 71-year-old female patient was referred to our center to upgrade a dual-chamber pacemaker (implanted in July 2019 for third-degree atrioventricular block) to a CRT-D system, following the detection of worsened systolic function [ejection fraction (EF): 25%–30%] at transthoracic echocardiogram. The patient had SIT with DXC and, in July 2019, had undergone mitral valve replacement (Carpentier Edwards type bioprosthesis no. 27; Edwards Lifesciences, Irvine, CA, USA) and tricuspid annuloplasty according to the Kay technique, with a concomitant left upper pulmonary lobectomy for neoplasm detected incidentally at cardiac tomography. During the preoperative assessment, a coronary angiography had been performed and revealed epicardial coronaries angiographically free from significant stenosis. Upon admission to our center, the electrocardiogram (ECG) showed sinus rhythm with a paced QRS duration of 140 ms and left bundle branch block morphology. At device check, the ventricular stimulation proportion was 98%. An additional transthoracic echocardiogram confirmed the low EF (25%). Despite optimal medical therapy, the patient remained in a poor functional class [New York Heart Association (NYHA) III].

**Figure 1:**
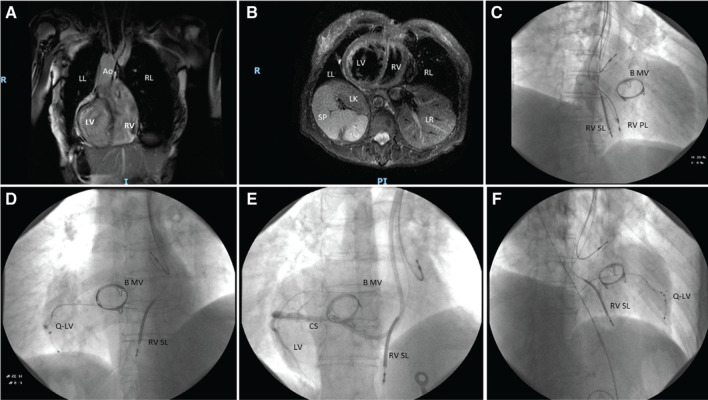
**A:** Cardiac MRI scan with long-axis view. **B:** Cardiac MRI scan in the short-axis view at papillary muscle sectioning. **C:** Anteroposterior view: dual-chamber pacemaker. **D:** CRT-D during “non-normalized” right anterior oblique view. **E:** Anteroposterior view coronary sinus during angiography. **F:** CRT-D during “normalized” left anterior view. B MV: biologic prosthetic mitral valve; CS: coronary sinus angiography; LL: left lung (bilobed lung); LR: liver; LV: left ventricle; Q-LV: quadripolar catheter in the left lateral vein; RK: right kidney; RL: right lung (trilobed lung); RV: right ventricle; RV PL: right ventricular pacing lead; RV SL: right ventricular shock lead; SP: spleen.

Taking into account the progression of negative remodeling, the severity of systolic dysfunction, and the amount of pacing, we decided to perform an upgrade to CRT-D (in January 2020) in accordance with current guidelines.^3^ Cardiac magnetic resonance imaging (MRI) performed in March 2019 had confirmed mirror-image DXC with no further abnormalities of the cardiac vascular structures observed **(Figures 1A and 1B)**. Through the existing scar over the left deltoid-pectoral grove, which was the site of the preexisting dual-chamber pacemaker, the left subclavian vein was accessed and a single-coil ventricular lead was placed in the apex of the RV **(Figure 1C)** and left ventricular (LV) lead in the lateral left vein **(Figure 1D)**, after confirmation of favorable coronary sinus (CS) anatomy with an intraprocedural venogram **(Figure 1E)**. The presence of a preexisting RV lead and the previous interventions of valve surgery facilitated the localization of the tricuspid valve and the CS cannulation.^4^ To ease our anatomic orientation during CS cannulation, we decided to flip the fluoroscopic image, obtained with a right anterior oblique view, by 180° (right–left), creating the optical effect of a levocardial position **(Figure 1F)**. The RV lead was extracted without complications applying a gentle simple traction without specialized extraction tools besides a standard stylet. The spontaneous interventricular RV-to-LV electrical delay was 110 ms. During RV pacing, the RV tip–to–LV tip delay was 130 ms. During LV pacing, the LV tip–to–RV tip delay was 110 ms. A CRT-D system (Resonate; Boston Scientific, Natick, MA, USA) was implanted using the preexisting dual-chamber pacemaker pocket on the left chest. The RV and LV pacing thresholds were 1 V and 2 V at a 0.4-ms duration. Postprocedural chest radiography confirmed correct positioning of the leads. The postprocedural course was marked by an absence of complications. Six months later, during an echocardiographic exam, the systolic function was improved (EF: 45%).

## Discussion

RV pacing is associated with pacemaker-induced cardiomyopathy. In a recent cohort, the incidence rate has been reported to be as high as 12.3% in those with high RV pacing burdens (> 20%) like as described in our case.^5^ Furthermore, there are major difficulties related to the interpretation of the anatomical reference points obtained with standard fluoroscopic projections, the inversion of eye/hand coordination of the operators, and the need for different manipulations of the devices commonly used, especially in the CS cannulation phase. In the present case, the possibility of adopting an epicardial approach was excluded due to the high risk correlated with the patient’s history of cardiac surgery.^6^ The rationale for using a left-sided venous approach was the presence of a previous device implanted in the left chest and the possibility of extracting the right ventricular lead, given the short lead dwelling time. During the procedure, to overcome problems due to unfamiliar hand/eye coordination and lead manipulation, we simply reversed the fluoroscopic image to recreate a standard levocardial orientation.^7^ This made RV lead implantation and CS cannulation easier to perform using standard tools. Moreover, the presence of a previously implanted pacemaker system facilitated the procedure that required a single intraprocedural venogram for the localization of CS.

## Conclusions

The present case shows that a right/left inversion of the fluoroscopic image simplifies the implantation procedure in DXC. Conventional CRT-D delivery systems can be used in these patients. The difficulties in performing invasive procedures in patients with DXC are related to congenital abnormalities of the cardiac structures; as such, reversing the fluoroscopic image recreates a standard levocardial orientation. This made RV lead implantation and CS cannulation easier to complete using standard tools. Moreover, the presence of a previously implanted pacemaker system facilitated the procedure that required a single intraprocedural venogram for localization of the CS. Given the possible presence of vascular abnormalities associated with DXC, a preimplantation assessment with computed tomography angiography or cardiac MRI can be helpful to detect congenital anatomic anomalies related to DXC.
